# Protective Effects of Oxytocin on Experimental Colitis: Modulation of Cytokines, Oxidative Stress, and Caspase-3 Activity

**DOI:** 10.3390/medicina62050893

**Published:** 2026-05-06

**Authors:** Burcu Aydinli Kazalak, Sibel Yaman, Mehmet Faik Ozcelik, Gozde Erkanli Senturk, Rahime Aydinli, Belisa Kaleci, Hafize Uzun

**Affiliations:** 1 Department of General Surgery, Cerrahpasa Faculty of Medicine, Istanbul University-Cerrahpasa, Istanbul 34098, Turkey; mfozcelik@gmail.com; 2 Department of General Surgery, Faculty of Medicine, İstanbul Medipol University, Istanbul 34214, Turkey; 3 Department of General Surgery, Gaziantep City Hospital, Gaziantep 27470, Turkey; syaman.ctf@hotmail.com; 4 Department of Histology and Embryology, Cerrahpasa Faculty of Medicine, Istanbul University-Cerrahpasa, Istanbul 34098, Turkey; gozde.erkanlisenturk@iuc.edu.tr; 5 Department of Anesthesia and Critical Care, Taksim Training and Research Hospital, Istanbul 34433, Turkey; rahimeaydinli78@gmail.com; 6 Department of Histology and Embryology, Ministry of Health, University Dental Clinic, 1001 Tiran, Albania; belisa.kaleci@klinikastomalogjikeuniversitare.gov.al; 7 Department of Medical Biochemistry, Faculty of Medicine, İstanbul Atlas University, Istanbul 34408, Turkey; hafize.uzun@atlas.edu.tr

**Keywords:** experimental colitis, oxytocin, 2,4,6-trinitrobenzene sulfonic acid, inflammation, antioxidant, apoptosis

## Abstract

*Background and Objectives*: This study investigated the effects of oxytocin (OT) in a 2,4,6-trinitrobenzenesulfonic acid (TNBS)-induced colitis model in rats, focusing on its potential anti-inflammatory, antioxidant, and anti-apoptotic properties. *Materials and Methods*: Forty male Wistar albino rats were randomly assigned to five groups (n = 8): control (intrarectal saline), TNBS-induced colitis, dexamethasone-treated colitis (1 mg/kg, i.p.), and two OT-treated colitis groups (0.5 and 1 mg/kg, i.p.). Body weight changes were recorded over 72 h. Macroscopic and histopathological evaluations were performed following laparotomy and colectomy. Serum and colon tissue samples were analyzed for TNF-α, IL-1β, IL-6, MPO, MDA, GSH, GPx, SOD, and CAT levels. Immunohistochemical analysis of caspase-3 and oxytocin receptor (OTR), as well as immunofluorescence-based OTR expression, were assessed. *Results:* OT treatment significantly improved macroscopic and histopathological findings compared with the colitis group (*p* < 0.01). Tissue TNF-α and MDA levels were reduced, while antioxidant parameters were generally improved in OT-treated groups. Caspase-3 immunoreactivity decreased, indicating reduced apoptosis. Although some changes were observed in IL-6 levels, these were not consistent across all comparisons. OTR immunoreactivity appeared reduced in colitis and partially restored following OT administration. *Conclusions*: OT attenuates experimental colitis by modulating inflammation, oxidative stress, and apoptosis, and may contribute to mucosal healing. These findings suggest that OT has potential as a supportive therapeutic agent in inflammatory bowel disease; however, further studies are required to clarify its mechanisms of action.

## 1. Introduction

Ulcerative colitis (UC) is an idiopathic inflammatory bowel disease characterized by recurrent inflammation of the colon and rectum, presenting with mucosal bleeding, ulceration, and acute inflammatory involvement. In UC, which is confined to the colon within the gastrointestinal tract, mucosal erosion and ulceration, edema in the mucosa and submucosa, and inflammatory cell infiltration are observed. T lymphocytes and macrophages are responsible for the resulting cellular damage [1].

Cytokines are molecules released from immune cells that play a key role in the development of inflammation and the generation of the immune response by increasing antigen-specific cell proliferation through autocrine, paracrine, and endocrine pathways [2,3,4]. In UC, the balance between anti-inflammatory and pro-inflammatory cytokines normally maintained under physiological conditions is disrupted due to immune hyperactivation. During this process, T helper 1 (Th1), Th2, regulatory T cells, antigen-presenting cells, Th17 cells, and various cytokines (tumor necrosis factor-α (TNF-α), interferon-γ, interleukin-1β (IL-1β, IL-6, IL-4, IL-5, IL-10, TGF-β, IL-13, IL-12, IL-18, IL-23) collectively contribute to both innate and adaptive immune responses. In UC, levels of pro-inflammatory cytokines, such as IL-1, IL-2, IL-6, IL-8, and TNF-α increase, whereas anti-inflammatory cytokines IL-4 and IL-10 decrease [2,3,4,5,6].

Oxytocin (OT), synthesized in the supraoptic and paraventricular nuclei of the hypothalamus, is a nanopeptide that has been shown in recent studies to exert anti-inflammatory effects by increasing neutrophil counts and inducing hyperalgesia [7]. Additionally, OT reduces the consumption of superoxide dismutase (SOD) and glutathione (GSH), and by potentially increasing nitric oxide (NO) levels, it inhibits apoptosis and inflammation through decreasing nicotinamide adenine dinucleotide phosphate oxidase and myeloperoxidase (MPO) enzyme activities [8,9,10]. It has also been demonstrated that OT influences mediators involved in inflammatory processes; that OT receptors contain elements sensitive to acute-phase proteins and interleukins; and that, through these mechanisms, OT likely reduces IL-6 secretion and exerts regulatory effects on the fibrinolytic system and coagulation [11,12,13].

Today, various agents are used to induce experimental colitis models. In our study, 2,4,6-trinitrobenzenesulfonic acid (TNBS) was preferred because this method produces a colitis model that closely resembles human inflammatory bowel disease (IBD) and induces colitis with a single administration [14,15,16]. In this study, we aimed to investigate the therapeutic efficacy and underlying mechanisms of OT, a peptide hormone with recognized immunomodulatory, antioxidant, and anti-apoptotic actions within a TNBS-induced experimental colitis model, to better clarify its potential role as a candidate treatment for inflammatory bowel disease.

## 2. Material and Methods

### 2.1. Experimental Procedure

Animal experiments were conducted at Istanbul University Aziz Sancar Experimental Medicine Research Institute with the approval of the Istanbul University Animal Experiments Ethics Committee (Date: 28 February 2020; Approval number: 2020/09). Wistar albino rats were housed in standard cages (four rats per cage) at 21 ± 1 °C under a 12 h light/dark cycle, with free access to standard chow and water under controlled temperature and humidity conditions.

Colitis was induced by intrarectal administration of 30 mg 2,4,6-trinitrobenzenesulfonic acid (TNBS) dissolved in 0.25 mL of 50% ethanol. This dose was selected based on established protocols demonstrating that it reliably produces reproducible, moderate-to-severe colitis within 72 h, closely mimicking the histopathological and inflammatory features of human ulcerative colitis without causing excessive mortality [17]. Colitis was induced using 30 mg of TNBS (120 mg/mL) dissolved in 0.25 mL of 50% ethanol and administered intrarectally. The TNBS solution was delivered to anesthetized rats positioned head-down on an inclined ramp by inserting an 8 cm catheter through the anal canal. During administration, the catheter was slowly withdrawn to ensure even distribution of the solution over the distal 8 cm of the colon rather than deposition at a single point. Rats were kept in this position briefly to prevent leakage. The same invasive procedures were applied to all groups to avoid procedural bias.

Forty male Wistar albino rats were randomly assigned to five groups. In the control group (Group 1, n = 8), physiological saline was administered intrarectally under anesthesia. Anesthesia was induced by intraperitoneal administration of ketamine (50–60 mg/kg) and xylazine (8–10 mg/kg). Following anesthesia, the rats were weighed and their body weights were recorded. Thirty minutes later, physiological saline was administered intraperitoneally once daily for 3 days. In the colitis group (Group 2, n = 8), physiological saline was administered intraperitoneally once daily for 3 days, starting 30 min after intrarectal TNBS administration. In the drug group (Group 3, n = 8), dexamethasone (1 mg/kg) was administered intraperitoneally once daily for 3 days, starting 30 min after TNBS administration. To evaluate the effects of OT, two OT-treated groups were included following TNBS administration: Group 4 (n = 8) received 0.5 mg/kg OT intraperitoneally once daily for 3 days, and Group 5 (n = 8) received 1 mg/kg OT intraperitoneally once daily for 3 days [9].

### 2.2. Macroscopical Assesment of Colitis

Seventy-two hours after the beginning of the experiment, laparotomy was performed under anesthesia via a midline incision in all rats, and the macroscopic appearance of the colon and surrounding tissues were examined. Macroscopic findings were evaluated according to previously published criteria [17]. Scoring of macroscopical damage is summarized, as shown in Table 1. Blood samples were collected from all animals, after which the rats were sacrificed. The colon was then removed for histopathological and biochemical analyses.

### 2.3. Histopathologic Analysis

Tissues were fixed in 10% neutral buffered formalin for 24 h, washed under running water for 5 h, and then processed through a graded alcohol series (70%, 80%, 90%, and 100%). Following dehydration, samples were cleared in toluene, kept in paraffin for 2 h, and subsequently embedded. Sections of 5 µm thickness were cut from the paraffin blocks using a microtome. These sections were used for histopathological and immunocytochemical evaluations. For histopathological examination, Hematoxylin and Eosin (H&E) staining was performed and evaluated under a light microscope. Scoring of histopathologic evaluation is summarized, as shown in Table 2 [18].

### 2.4. Immunocytochemical Analysis

The OT dose used in this study was determined based on the dosing regimen reported in a stress-aggravated colitis model in rats, where the protective effects of OT and the role of OT receptors were demonstrated [9]. Immunocytochemical analysis was performed using OT receptor (OTR Antibody, Santa Cruz Biotechnology, Dallas, TX, USA) and caspase-3 (Caspase-3 Antibody, Santa Cruz Biotechnology, Dallas, TX, USA) antibodies. After deparaffinization of the tissues, dehydration was completed, followed by washing in PBS. Endogenous peroxidase activity was blocked by incubating the sections in peroxidase solution for 5 min. The samples were then washed in TBS (3 × 5 min), and blocking was performed according to the HRP kit protocol (UltraVision Polyvalent, Rabbit–Mouse, THERMO, Rockford, IL, USA). Antibody dilutions were prepared according to the manufacturer’s instructions: OT receptor at 1:200 and caspase-3 at 1:250. Sections were incubated with primary antibodies overnight at +4 °C. After incubation, sections were washed in T-PBS, followed by application of the secondary antibody and HRP. In the final step, AEC chromogen was used for visualization. All sections were examined and photographed using an Olympus photomicroscope. Immunohistochemical staining was also evaluated semi-quantitatively using the H-score method, which incorporates both the intensity and percentage of positively stained cells, allowing comparative assessment across groups.

### 2.5. Immunofluorescence Method

The oxytocin receptor (OTR) was also evaluated using the immunofluorescence method. Antigen retrieval was performed using Tris-EDTA buffer (pH 9.0). The tissues were washed with PBS and then blocked with 5% BSA for 1 h to prevent non-specific background staining. Sections were incubated with the primary anti-OTR antibody (same brand as used in the immunohistochemistry procedure) and kept at +4 °C overnight. The following day, the tissues were washed with PBS to remove the primary antibody and then incubated with Alexa Fluor 488–conjugated secondary antibody. After a final PBS wash, the sections were mounted with a medium containing DAPI for nuclear staining. The samples were examined and micrographed using a fluorescence microscope (Olympus BX61, Tokyo, Japan).

### 2.6. Evaluation of OTR and Caspase-3 by Immunohistochemistry

Immunohistochemical staining for OTR and caspase-3 was semi-quantitatively evaluated using the histological score (H-score) method.

### 2.7. Biochemical Assessments

Tissues were washed in cold phosphate buffer (0.01 M, pH 7.4) immediately after collection. After weighing, the tissue samples were minced and homogenized in phosphate buffer at +4 °C using a glass homogenizer to obtain 10% (*w*/*v*) homogenates. The homogenates were then centrifuged at 5000× *g* for 5 min in a refrigerated Eppendorf centrifuge, and biochemical analyses were performed using the resulting supernatants. Supernatants were stored at −80 °C until analysis.

The levels of TNF-α, IL-1β, IL-6, MPO, MDA, GSH, GPx, SOD, and CAT activity were measured by ELISA using commercially available kits (BT-LAB, Bioassay Technology, Shanghai, China) in accordance with the manufacturer’s instructions. All samples were analyzed in duplicate following the provided protocols. Intra- and inter-assay coefficients of variation for all tests were <8%.

### 2.8. Statıstıcal Analysis

All statistical analyses were performed using IBM SPSS Statistics for Windows, Version 29.0.2.0. The normality of data distribution was assessed using the one-sample Kolmogorov–Smirnov test. As the data did not meet the assumptions for parametric testing, non-parametric methods were applied. Comparisons between groups were performed using the Kruskal–Wallis test. When a statistically significant difference was detected, pairwise comparisons were conducted using the Mann–Whitney U test to identify the source of the difference. Categorical variables were analyzed using the chi-square test. Continuous variables are presented as median (interquartile range, IQR), in accordance with the non-parametric distribution of the data (mean ± standard deviation is also provided in tables for completeness). To ensure consistency, results in the text are reported using median (IQR), while both mean ± SD and median (IQR) are presented in tables for completeness. A *p*-value < 0.05 was considered statistically significant. No formal sample size calculation or power analysis was performed, as the study was designed as an experimental exploration study.

## 3. Results

### 3.1. Macroscopic Results

Macroscopic scores differed significantly between groups (*p* < 0.001). The control group showed lower scores compared to the colitis, dexamethasone, and 0.5 mg/kg OT groups. The 1 mg/kg OT group also demonstrated significantly lower scores compared to the colitis, dexamethasone, and 0.5 mg/kg OT groups (*p* < 0.05), indicating reduced macroscopic damage (Table 3).

### 3.2. Histopathologic Results

Histopathological evaluation revealed clear differences among the groups. The control group exhibited normal histological architecture in the mucosa, submucosa, and muscular layers, with no evidence of epithelial or lamina propria damage. In contrast, the colitis group showed severe epithelial disruption, marked inflammation, goblet cell depletion, and pronounced glandular destruction within the lamina propria. Submucosal edema, inflammation, and hemorrhage were also evident, along with vacuolization and structural damage in the muscularis layer.

**Figure 1:**
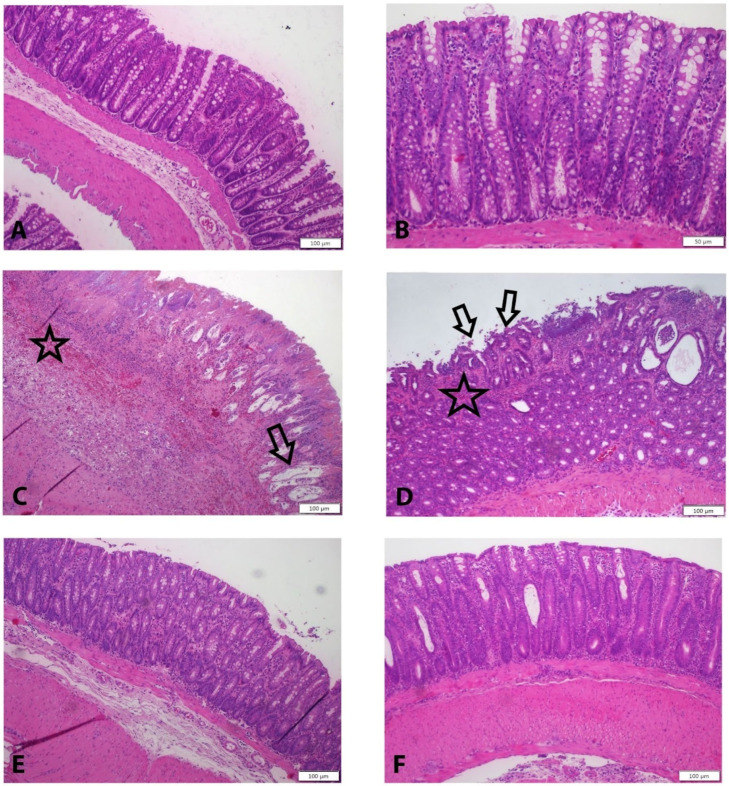
(**A**) Regular morphology of mucosa, submucosa, and muscular layers were seen in the control group; (**B**) normal morphology of epithelium, lamina propia, and glandular structures were shown in the control group; (**C**) severe damage was demonstrated in all layers in the colitis group; (**D**) damage of mucosal layer were seen in the Dexa group; and (**E**) less damage were observed in 0.5 OT and (**F**) 1 OT groups. H&E Staining, Scale bar: (**A**,**C**–**F**): 50 µm, (**B**): 20 µm. Arrow: mucosal damage, star: hemorrhage.

In the dexamethasone group, epithelial damage and inflammation were present but less severe compared to the colitis group, with focal inflammatory areas and mild submucosal edema. Both OT-treated groups demonstrated further improvement, characterized by reduced epithelial damage and preservation of goblet cell morphology. Submucosal edema was mild in the 0.5 mg/kg OT group and nearly absent in the 1 mg/kg OT group. Similarly, vacuolization in the muscularis layer was observed in the 0.5 mg/kg OT group but was markedly reduced at the higher dose (Figure 1, Table 4).

### 3.3. Caspase-3 Immunohistochemistry Results

Caspase-3 immunoreactivity showed distinct distribution patterns across the groups. In the control group, caspase-3-positive cells were observed in surface and glandular epithelial cells as well as in stromal cells. In contrast, the colitis group exhibited widespread caspase-3 expression across all intestinal layers, including the myenteric (Auerbach) plexus, indicating increased apoptotic activity.

**Figure 2:**
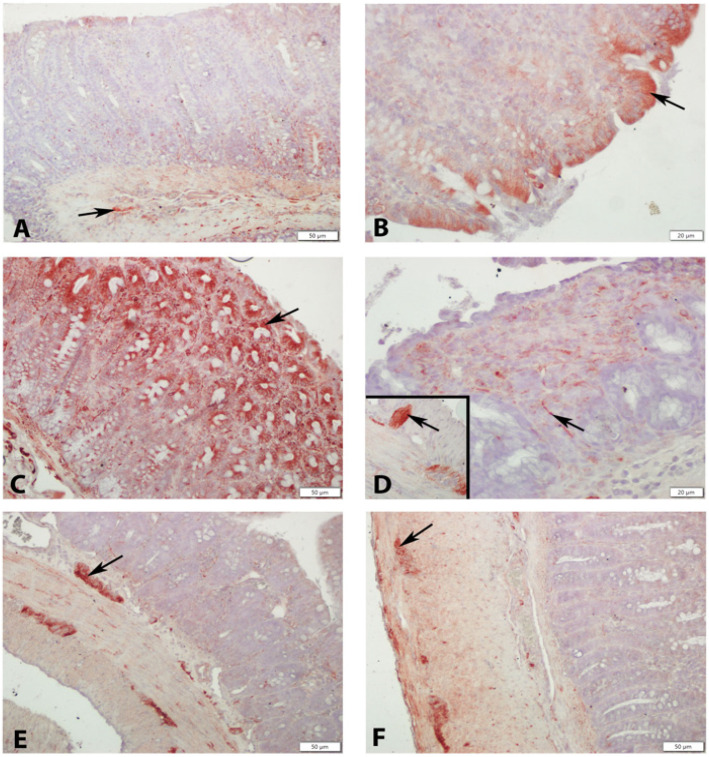
(**A**,**B**) Few Caspase 3 ir (+) cells (arrows) observed in the control group. (**C**) Numerous cells with Caspase 3 ir (+) observed in the colitis group. (**D**) Caspase 3 ir (+) cells in the mucosa seen in the Dexa group (Inset: Caspase 3 ir (+) cells in the myenteric plexus (arrows)). (**E**) Caspase 3 ir (+) cells (arrows) observed in 0.5 OT and (**F**) 1 OT groups. Caspase 3 immunohistochemistry, scale bar: (**A**,**C**,**E**,**F**): 50 µm, (**B**,**D**): 20 µm.

In the dexamethasone group, caspase-3-positive cells were mainly localized to areas with structural damage and within the myenteric plexus. Both OT-treated groups demonstrated a marked reduction in caspase-3 immunoreactivity, with only occasional positive cells observed in limited regions and in the myenteric plexus (Figure 2, Table 5).

### 3.4. Oxytocin Receptor (OTR) Immunohistochemistry and Immunofluorescence Results

**Figure 3:**
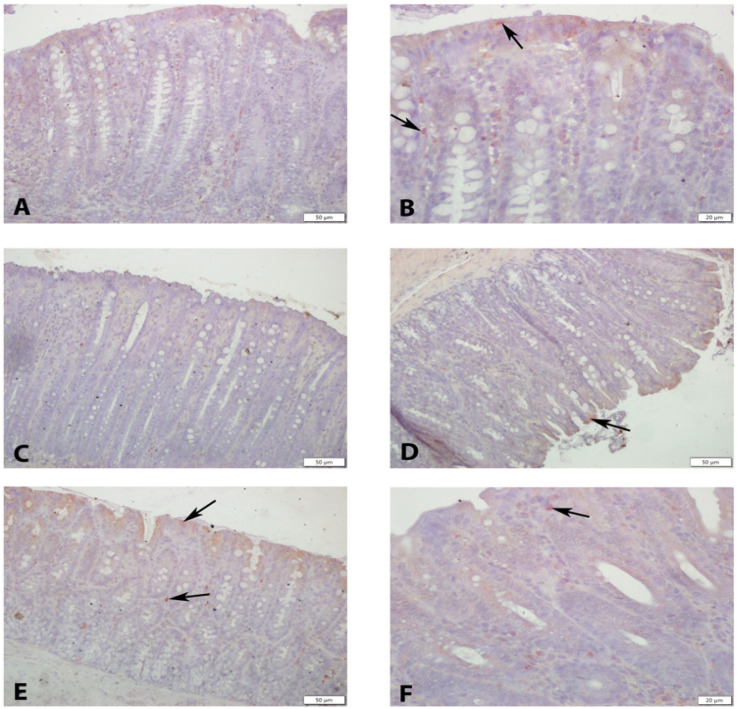
(**A**,**B**) OTR ir (+) cells (arrows) observed in the control group. (**C**) Cells with OTR ir (+) observed in the colitis group. (**D**) OTR ir (+) cells in the mucosa seen in the Dexa group. (**E**) OTR ir (+) cells (arrows) observed in 0.5 OT and (**F**) 1 OT groups. OTR immunohistochemistry, scale bar: (**A**,**C**,**E**,**F**): 50 µm, (**B**,**D**): 20 µm.

**Figure 4:**
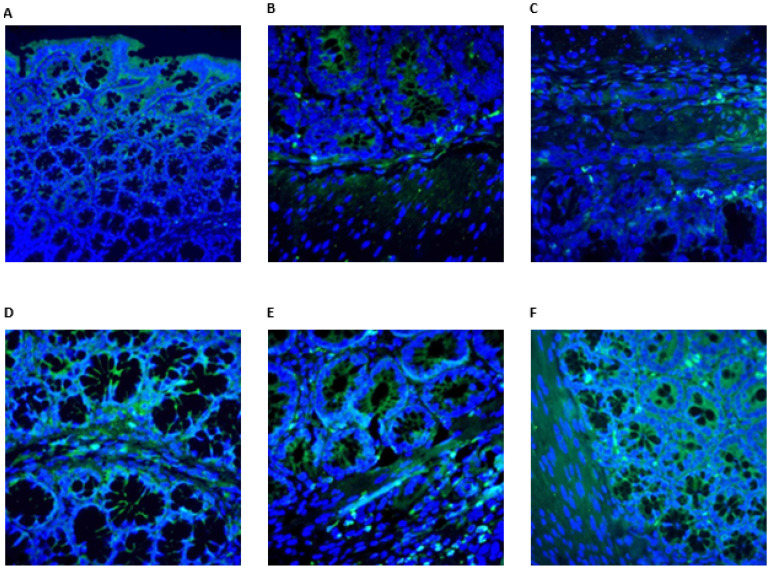
(**A**) OTR expression (+) in surface epithelium. (**B**) OTR expression (+) cells both mucosa and muscle layers observed in the control group. (**C**) OTR expression (+) cells in stromal cells seen in the colitis group. (**D**) OTR expression (+) cells in the mucosa observed in the Dexa group. (**E**) OTR expression (+) cells both mucosa and muscle layer in 0.5 OT and (**F**) 1 OT groups. Green fluorochrome: OTR; blue fluorochrome: DAPI (cell nuclei).

OTR immunoreactivity showed distinct distribution patterns among the groups. In the control group, OTR expression was detected in the apical cytoplasm of surface epithelial cells, glandular epithelium, and in selected cells of the lamina propria, submucosa, and muscular layer, smooth muscle and plexus cells. In the colitis group, OTR expression was markedly reduced, with an absence of immunoreactivity in damaged epithelial regions. Weak staining was observed only in stromal and smooth muscle cells outside the affected areas. In the dexamethasone group, OTR immunoreactivity was present in the surface and glandular epithelium, as well as in smooth muscle and stromal cells, predominantly in regions associated with tissue damage. Both OT-treated groups demonstrated preserved OTR expression in the mucosal epithelium, glandular structures, and muscular layer, indicating a relative restoration compared to the colitis group (Figure 3 and Figure 4).

### 3.5. Results of Oxytocin and Caspase-3

OTR and caspase-3 expression levels across the groups are summarized in Table 5. Caspase-3 levels were significantly lower in the control group compared to the colitis and 0.5 mg/kg OT groups (*p* = 0.001 and *p* = 0.012, respectively). In contrast, the colitis group exhibited significantly higher caspase-3 levels than the dexamethasone and both OT-treated groups (*p* < 0.01). The dexamethasone group showed lower caspase-3 levels compared to both OT-treated groups (*p* < 0.05), while the 0.5 mg/kg OT group had higher levels than the 1 mg/kg OT group (*p* < 0.05), indicating a dose-dependent reduction with higher OT administration (Table 5).

### 3.6. Results of Serum Biochemical Parameter Analysis

Serum biochemical parameters are summarized in Table 6. No significant differences were observed in TNF-α and IL-1β levels between groups (*p* > 0.05). In contrast, serum IL-6 levels differed significantly (*p* < 0.05), with lower levels in the colitis group compared to the control and 1 mg/kg OT groups. MPO levels were significantly lower in the colitis group than in the dexamethasone and both OT-treated groups (*p* < 0.01), while the control group also exhibited lower MPO levels compared to the 1 mg/kg OT group (*p* < 0.05). MDA levels were significantly reduced in both OT-treated groups compared to the control, colitis, and dexamethasone groups (*p* < 0.01). No significant differences were found in GSH levels (*p* > 0.05). Antioxidant parameters showed improvement with OT treatment: GPx levels were significantly lower in the colitis and dexamethasone groups compared to both OT-treated groups (*p* < 0.01), and SOD activity was also reduced in these groups compared to OT-treated groups (*p* < 0.05). Serum CAT activity was highest in the 1 mg/kg OT group, showing significant increases compared to all other groups (*p* < 0.001). Overall, OT treatment, particularly at the higher dose, was associated with improved antioxidant status and reduced oxidative stress markers.

### 3.7. Results of Tissue Biochemical Parameter Analysis

As shown in Table 7, significant differences were observed among groups in several tissue inflammatory and oxidative stress parameters. TNF-α levels were significantly lower in the 1 mg/kg OT group compared to the control, colitis, and dexamethasone groups (*p* = 0.043). In contrast, IL-6 levels were significantly higher in the dexamethasone group than in both OT-treated groups (*p* = 0.020). MPO levels were significantly reduced in the colitis group compared to the control and OT-treated groups (*p* = 0.009).

MDA levels were significantly decreased in both OT-treated groups compared to the control, colitis, and dexamethasone groups (*p* = 0.001). Antioxidant markers showed improvement with OT treatment: GSH and GPx levels were higher in the OT-treated groups, particularly at the higher dose (*p* = 0.045 and *p* = 0.042, respectively). CAT activity was increased in the dexamethasone group and was also higher in the 1 mg/kg OT group compared to the colitis group (*p* = 0.004). No significant differences were observed for IL-1β and SOD levels (*p* > 0.05).

Overall, OT treatment, especially at the higher dose, was associated with reduced oxidative stress and improved antioxidant status in colon tissue; however, these findings should be interpreted within the experimental context.

## 4. Discussion

The most important findings of this study demonstrate that OT exhibits a significant therapeutic effect in the TNBS-induced colitis model. OT administration, particularly at a dose of 1 mg/kg, significantly improved macroscopic and histopathological colitis scores by reducing epithelial damage, goblet cell loss, submucosal edema, and hemorrhage. The significant decrease in TNF-α at the tissue level supports the potent local anti-inflammatory effect of OT, while the decrease in serum and tissue MDA levels and the increase in antioxidant enzyme activities such as CAT, SOD, GPx, and GSH demonstrated a marked antioxidant protective effect. Furthermore, the significant decrease in caspase-3-positive cells in the OT groups confirmed the anti-apoptotic effect of OT. OT receptors were found to be reduced in colitis but increased again in the epithelium, stromal cells, and smooth muscle layer with OT administration, demonstrating that OT supports tissue healing through receptor-mediated mechanisms. It is widely recognized in the literature that the OTR is primarily located in smooth muscle cells. In our study, in addition to these established findings, we demonstrated for the first time the presence of the OTR in the colonic surface and glandular epithelium. All these findings indicate that OT is a strong candidate for the treatment of ulcerative colitis due to its effects of suppressing inflammation, reducing oxidative stress, inhibiting apoptosis, and preserving mucosal barrier integrity.

The present study aimed to investigate the therapeutic potential of OT, a peptide with well-documented anti-inflammatory, antioxidant, and anti-apoptotic properties, in a TNBS-induced experimental colitis model. OT immunoreactivity has been reported in the myenteric plexuses of the stomach, ileum, proximal and distal colon, and in the submucosal plexuses of the stomach, where it regulates gastrointestinal motility, smooth muscle relaxation, water-electrolyte absorption, and enteric neuronal activity. In animal models lacking OTR, increased intestinal emptying, severe colitis, shortened crypts, and decreased villus proliferation have been observed, highlighting its role as a regulator of the brain–gut axis and intestinal homeostasis [19,20,21,22].

### 4.1. Experimental Model and Rationale

The TNBS-induced colitis model was chosen for its similarity to human UC, characterized by weight loss, bloody diarrhea, increased colonic wall thickness, transmural inflammation, infiltration of submucosal T lymphocytes, macrophages, and granulocytes, goblet cell loss, diffuse necrosis, and altered cytokine profiles [23,24,25,26]. Disease severity ranges from mild mucosal lymphocytic infiltration to extensive transmural inflammation with crypt loss, crypt abscesses, irregular mucosal surface, and hemorrhage. This model allows the assessment of therapeutic interventions in a controlled environment that closely reflects the pathophysiology of UC.

### 4.2. Histopathological and Macroscopic Findings

In the pathophysiology of UC, an often incompletely understood but crucial component is the mucus layer covering epithelial cells. Disruption of the mucus layer, which exhibits antimicrobial properties released by goblet cells, between the epithelial layer and intestinal microbiota can contribute to protective activity against pathogens and commensal inflammations. Dysfunction in autophagy in goblet cells, reducing the mucus layer, is believed to contribute to the development of UC by compromising barrier integrity. Some studies have associated this condition with decreased goblet cell numbers and reduced secretion response, leading to the breakdown of the normally impermeable mucus layer, allowing bacterial penetration and the development of colitis in active UC patients [19,24,27,28,29].

In this study, administration of 1 mg/kg OT significantly reduced both macroscopic and histopathological colitis scores compared to the colitis and dexamethasone groups. OT treatment preserved epithelial integrity, minimized goblet cell loss, and reduced submucosal edema, hemorrhage, and muscular layer vacuolization. These improvements were dose-dependent, with the 1 mg/kg group showing more pronounced effects than the 0.5 mg/kg group, indicating that OT promotes mucosal healing and mitigates tissue injury in colitis. Histopathological examination using H&E staining supported these findings. In the colitis group, severe inflammation was observed in the mucosal layer, accompanied by intense epithelial damage and reduced goblet cell numbers. The submucosal layer exhibited edema, hemorrhage, and significant vascular and muscular damage. In the dexamethasone-treated group, epithelial damage was less pronounced, and submucosal edema persisted to a limited extent. In OT-treated groups, minimal epithelial damage was observed, and goblet cells appeared normal. Submucosal edema was partially present in the 0.5 mg/kg OT group but was nearly absent in the 1 mg/kg group. Vacuolization in the muscular layer was also less pronounced in the 1 mg/kg group. Overall, OT administration demonstrated a dose-dependent and significant improvement in histopathological parameters of colitis.

Recent reports have raised concerns regarding the specificity of some commercially available OTR antibodies in different tissues and experimental conditions [21,30]. Therefore, OTR immunostaining findings in the present study should be interpreted cautiously. In this study, OTR evaluation was used primarily as a morphological localization tool rather than definitive evidence of receptor-specific signaling. Negative control sections were included to minimize non-specific staining, and all groups were processed under identical conditions. Importantly, the main evidence for the protective effects of OT relies on consistent histopathological improvement, cytokine modulation, oxidative stress parameters, and reduced caspase-3 activity, while OTR staining is presented as supportive observational data rather than proof of direct receptor-mediated mechanisms.

Recent reports have raised concerns regarding the specificity of some commercially available OTR antibodies in different tissues and experimental conditions. Therefore, OTR immunostaining findings in the present study should be interpreted cautiously. In this study, OTR evaluation was used primarily as a morphological localization tool rather than definitive evidence of receptor-specific signaling. Negative control sections were included to minimize non-specific staining, and all groups were processed under identical conditions. Importantly, the main evidence for the protective effects of OT relies on consistent histopathological improvement, cytokine modulation, oxidative stress parameters, and reduced caspase-3 activity, while OTR staining is presented as supportive observational data rather than proof of direct receptor-mediated mechanisms. Although OT administration was associated with significant improvements in histopathological findings, cytokine profiles, oxidative stress parameters, and apoptosis markers in the TNBS-induced colitis model, the present study design does not allow definitive conclusions regarding direct causal mechanisms. These biochemical and cellular changes should be interpreted as accompanying biological responses correlated with mucosal healing rather than proof of direct receptor-mediated or pathway-specific effects. Therefore, OT’s therapeutic role in this model is best described as being associated with anti-inflammatory, antioxidant, and anti-apoptotic profiles that may contribute to tissue protection.

### 4.3. Inflammatory and Cytokine Profiles

In UC, pro-inflammatory cytokines such as IL-1, IL-2, IL-6, IL-8, and TNF-α are typically elevated, while anti-inflammatory cytokines like IL-4 and IL-10 are decreased. IL-1 and TNF-α exacerbate mucosal inflammation and damage the epithelial barrier [24]. In our study, although serum TNF-α and IL-1β levels did not differ significantly between groups, tissue TNF-α levels were significantly lower in the 1 mg/kg OT group compared to both the control and colitis groups, indicating a strong local anti-inflammatory effect of OT. Tissue IL-1β levels, however, remained similar across groups. Interestingly, although serum IL-6 levels are generally expected to increase in ulcerative colitis, they were lower in the colitis group compared to the control and 1 mg/kg OT groups. This unexpected observation may be related to factors such as the timing of sample collection, heterogeneity in disease stage, or the involvement of compensatory regulatory mechanisms. Therefore, these findings should be interpreted with caution. In contrast, tissue IL-6 levels were significantly reduced in the OT-treated group compared to the dexamethasone group, suggesting a potential modulatory effect of OT on the local inflammatory response. However, these results should be considered within the limitations of the experimental model. These findings may indicate that OT contributes to the regulation of the local inflammatory milieu and may support mucosal integrity and reduce epithelial apoptosis [24]. Cytokine responses in experimental and clinical IBD are highly dynamic and may vary depending on disease stage, tissue compartment, and timing of measurement, which may account for discrepancies such as IL-6 variability.

### 4.4. Oxidative Stress and Antioxidant Effects

OT also mitigated oxidative stress by decreasing serum and tissue MDA levels and enhancing endogenous antioxidant defenses, including CAT, SOD, GPx, and GSH, supporting its dual role in reducing oxidative injury and inflammation [19,29,31,32]. In UC, oxidative stress induces activation of antioxidant enzymes such as GPx, MPO, and SOD in plasma and colon mucosa, while levels of antioxidant substances like vitamins C and E, selenium, GSH, and β-carotene tend to decrease, reflecting the close link between oxidative stress and inflammation [28]. In colitis models, MPO levels and MDA, an indicator of lipid peroxidation and tissue damage, are elevated, while reductions in SOD and GSH demonstrate oxidative imbalance and apoptotic activity [29,31,32].

In our study, serum MPO levels were higher in the 0.5 and 1 mg/kg OT and dexamethasone groups than in the colitis group, whereas tissue MPO levels were lower in colitis compared to OT-treated groups. Serum and tissue MDA levels were significantly reduced by OT treatment, indicating protection against tissue damage. Serum CAT activity and tissue CAT activity were significantly higher in the 1 mg/kg OT group compared to all other groups, suggesting enhanced antioxidant capacity. Serum SOD levels were elevated in both OT-treated groups, with the 1 mg/kg group showing the highest tissue SOD activity. Although serum GSH levels did not differ significantly, tissue GSH levels were higher in the 1 mg/kg OT group compared to colitis. Finally, GPx activity in both serum and tissue was significantly increased in OT-treated groups. These findings confirm the antioxidant, anti-inflammatory, and tissue-protective effects of OT in experimental colitis.

### 4.5. OT Receptor Expression and Anti-Apoptotic Effects

Caspases, particularly caspase-3, are proenzyme-like proteases that mediate apoptotic cell death, promote cytokine maturation, and contribute to fibrosis through myofibroblast differentiation [32,33,34,35]. Caspase-3 can also induce pyroptosis, further exacerbating inflammation in colitis and other diseases [34]. In colitis models, caspase-3 expression is elevated, and its reduction protects the intestinal epithelium by limiting apoptosis and pyroptosis [33,34,36]. In this study, OTR expression, which was diminished in colitis-affected tissues, was restored in OT-treated groups across epithelial surfaces, smooth muscle, and plexus cells. Caspase-3 immunoreactivity, a marker of apoptosis, was markedly elevated in colitis but significantly reduced in OT-treated groups, confirming anti-apoptotic effects and preservation of mucosal integrity [32,33,34,35,37]. Elevated tissue OT levels correlated with improved histopathology and goblet cell preservation, highlighting a receptor-mediated protective mechanism. Intense caspase-3 (ir) positivity was observed in all layers, including myenteric and Auerbach plexuses, in the colitis group. Dexamethasone treatment reduced caspase-3 expression in damaged areas, while OT treatment further decreased caspase-3 levels in a dose-dependent manner, with the 1 mg/kg group showing greater reduction than the 0.5 mg/kg group, confirming the tissue-protective effects of OT. Experimental studies have demonstrated that OT is protective in gastrointestinal injury and colitis models [38]. OT and OTR are widely expressed by intestinal cells, particularly in the myenteric plexus of the colon [39,40].

OTR-positive neurons are found in the submucosal and myenteric plexuses of the stomach, jejunum, ileum, and colon [40], and OTR expression in intestinal epithelial cells stabilizes adherens junctions and preserves enterocyte integrity [39]. Functionally, the OT/OTR system modulates intestinal motility through intrinsic primary afferent neurons, present on approximately three-quarters of myenteric neurons. OT converts physiological stimuli, including villus movement, intestinal muscle contraction, and luminal chemical changes, into neuronal activity, reducing mucosal activation and supporting enteric neuronal survival. In the absence of OTR, intestinal motility increases, crypt depth decreases, luminal absorption is impaired, and fecal output rises, enhancing susceptibility to inflammation [21,41,42,43]. In the present study, OTR immunoreactivity (ir) was observed in the apical cytoplasm of surface epithelial cells, lamina propria, some submucosal cells, glandular epithelial cytoplasm, smooth muscle, and plexus cells in control tissue. In contrast, OTR expression was absent in damaged areas of the colitis group, with only weak staining in stromal and muscle cells outside lesions. Dexamethasone treatment partially restored OTR expression, whereas OT-treated groups exhibited robust OTR positivity in mucosal surfaces, glandular epithelium, smooth muscle, and stromal cells. Tissue OT levels were significantly higher in OT-treated groups compared with colitis, with the 0.5 mg/kg group showing higher levels than the 1 mg/kg and Dexa groups, suggesting that exogenous OT enhances receptor expression in damaged tissues. Recent reports have raised concerns regarding the specificity of some commercially available OTR antibodies [30]. Therefore, OTR immunostaining findings in the present study should be interpreted with caution. In this context, OTR expression was evaluated primarily for morphological localization rather than as definitive evidence of receptor-specific signaling. Accordingly, the observed protective effects of oxytocin are based mainly on histopathological, biochemical, and apoptotic findings, and do not establish direct receptor-mediated mechanisms.

### 4.6. Limitations of Study

The absence of macroscopic assessment of colon length represents a limitation of the present study. The selected oxytocin doses (0.5 and 1 mg/kg) were based on previous experimental studies demonstrating anti-inflammatory and protective effects in similar animal models. The study design included a saline-treated control group, a TNBS-induced colitis group, and a dexamethasone-treated positive control, consistent with standard experimental colitis models; however, the lack of additional control groups (e.g., oxytocin-only or vehicle-treated groups) should be considered a limitation. In addition, multiple comparisons were not adjusted, which may increase the risk of type I error and should be considered when interpreting the findings. Although semi-quantitative evaluation using H-score was performed for OTR and caspase-3 expression, the absence of fully quantitative image-based analysis represents another limitation. Furthermore, the TNBS model reflects an acute, chemically induced form of colitis and does not fully capture the chronic and heterogeneous nature of human inflammatory bowel disease, which should be considered when extrapolating these results.

## 5. Conclusions

The relatively small sample size, short experimental duration, and the absence of functional outcome assessments (e.g., clinical or behavioral parameters) represent further limitations and should be considered when interpreting the results. OT attenuated TNBS-induced colitis in this experimental model, as evidenced by reductions in inflammatory, oxidative stress, and apoptotic markers, along with preservation of epithelial integrity and goblet cell function. These findings suggest that OT may contribute to the modulation of mucosal injury and support intestinal barrier function. Variations observed in cytokine levels, particularly IL-6, may reflect the dynamic and context-dependent nature of inflammatory responses, influenced by factors such as disease stage and timing of sampling. However, these results are based on associative findings and do not establish definitive mechanistic or receptor-mediated effects. Therefore, OT may be considered a potential adjunctive therapeutic candidate in inflammatory bowel disease, although further experimental and clinical studies are required to confirm its efficacy and clarify its underlying mechanisms. The present findings are based primarily on histopathological and biochemical observations and do not directly elucidate the underlying molecular mechanisms. Therefore, the observed effects of oxytocin should be interpreted as associative rather than causal, and further mechanistic studies are required.

## Figures and Tables

**Table 1:** Scoring of macroscopical damage [17].

	Normal Mucosa
1	Localized hyperemia, no ulcer
2	Linear ulcer without obvious inflammation
3	Linear ulcer with inflammation in a single area
4	Two or more areas of inflammation and/or ulceration
5	An ulcer longer than 1 cm accompanied by two or more major areas of inflammation and ulceration or inflammation in a single area of the colon

**Table 2:** Scoring of histopathologic evaluation [18].

Score	Loss of Mucosal Structure	Inflamatory Cell Infiltration	Muscular Thickening	Cryptic Abscess	Goblet Cell Depletion
0	absent	absent	absent	absent	absent
1	mild	mild	mild	available	available
2	modarate	modarate	modarate	-	-
3	severe	severe	severe	-	-

**Table 3:** Macroscopical evaluations according to groups.

		Mean ± Std	Median (IQR)	*p*	Post Hoc
**Macroscopical evaluation**	**^1^ Control**	0 ± 0	0 (0–0)	0.001 **	**1 < 2, 3, 4**
	**^2^ Colitis**	4.12 ± 1.35	4.5 (1–5)		
	**^3^ Dexa**	3.63 ± 1.68	4 (1–5)		
	**^4^ 0.5 OT**	3.25 ± 1.83	4 (0–5)		
	**^5^ 1 OT**	0.88 ± 0.65	1 (0–2)	0.05 *	**5 < 2, 3, 4**

* *p* < 0.05 = 1 OT vs. Colit, Dexa, 0.5 OT; ** *p* < 0.01 = Control vs. Colit, Dexa, 0.5 OT. Kruskal–Wallis test & post hoc Dunn test. Numbers in the Post Hoc column refer to group labels: ^1^ = Control, ^2^ = colitis, ^3^ = Dexamethasone, ^4^ = OT 0.5 mg/kg, ^5^ = OT 1 mg/kg Symbols ‘<’ and ‘>’ indicate statistically significant differences between groups based on Dunn’s post hoc test.

**Table 4:** Evaluations of inflammation, epithelial damage, glandular damage, and hemorrhage of histopathologic scores according to groups.

		Mean ± Std	Median (IQR)	*p*	*Post Hoc*
**Inflammation**	**^1^ Control**	0 ± 0	0 (0–0)	0.001 **	***1 < 2, 3, 4, 5***
	**^2^ Colitis**	2.25 ± 0.65	2.3 (2–2.75)		***2 > 5***
	**^3^ Dexa**	1.84 ± 0.90	1.6 (1–2.75)		
	**^4^ 0.5 OT**	1.75 ± 0.76	1.8 (1–2.25)		
	**^5^ 1 OT**	1.06 ± 0.44	1 (0.875–1.125)		
**Epithelial damage**	**^1^ Control**	0.19 ± 0.26	0 (0–0.5)	0.001 **	***1 < 2, 3, 4, 5***
	**^2^ Colitis**	1.69 ± 0.84	2 (1.25–2.25)		
	**^3^ Dexa**	1.75 ± 1.00	1.5 (1–2.75)		
	**^4^ 0.5 OT**	1.41 ± 0.83	1 (0.75–2)		
	**^5^ 1 OT**	1.06 ± 0.69	0.9 (0.5–1.75)		
**Glandular damage**	**^1^ Control**	0.06 ± 0.18	0 (0–0)	0.001 **	***1 < 2, 3, 4, 5***
	**^2^ Colitis**	2.09 ± 0.42	2 (2–2.125)		***2 > 5***
	**^3^ Dexa**	1.69 ± 0.96	1.5 (1–2.5)		
	**^4^ 0.5 OT**	1.44 ± 0.82	1 (1–2)		
	**^5^ 1 OT**	1.00 ± 0.55	0.9 (0.5–1.375)		
**Hemorrhage**	**^1^ Control**	0.06 ± 0.18	0 (0–0)	0.001 **	***1 < 2, 3, 4, 5***
	**^2^ Colitis**	2.09 ± 0.42	2 (2–2.125)		***2 > 5***
	**^3^ Dexa**	1.69 ± 0.96	1.5 (1–2.5)		
	**^4^ 0.5 OT**	1.44 ± 0.82	1 (1–2)		
	**^5^ 1 OT**	1.00 ± 0.55	0.9 (0.5–1.375)		

Kruskal–Wallis test & post hoc Dunn test ** *p* < 0.01. Numbers in the Post Hoc column refer to group labels: ^1^ = Control, ^2^ = colitis, ^3^ = Dexamethasone, ^4^ = OT 0.5 mg/kg, ^5^ = OT 1 mg/kg Symbols ‘<’ and ‘>’ indicate statistically significant differences between groups based on Dunn’s post hoc test.

**Table 5:** H-score evaluation of OTR and caspase-3 by immunohistochemistry.

		Mean ± Std	Median (IQR)	*p*	*Post Hoc*
**Oxytocin Receptor (%)**	**^1^ Control**	13.00 ± 2.12	13 (10–15)	0.005 **	***1 > 2, 3***
	**^2^ Colitis**	5.10 ± 2.19	5 (2–8)		***2 < 4, 5***
	**^3^ Dexa**	6.40 ± 6.42	8 (0–15)		***3 < 4***
	**^4^ 0.5 OT**	16.40 ± 6.10	15 (10–25)		***5 < 4***
	**^5^ 1 OT**	8.80 ± 2.39	10 (5–11)		
**Caspase-3 (%)**	**^1^ Control**	25.60 ± 4.83	25 (20–31)	0.001 **	***1 < 2, 4***
	**^2^ Colitis**	85.00 ± 5.00	85 (80–90)		***2 > 3, 4, 5***
	**^3^ Dexa**	23.40 ± 4.22	22 (20–30)		***3 < 4, 5***
	**^4^ 0.5 OT**	48.40 ± 2.70	48 (45–52)		***4 > 5***
	**^5^ 1 OT**	40.00 ± 3.54	40 (35–45)		

Kruskal–Wallis test & post hoc Dunn test ** *p* < 0.01. Numbers in the Post Hoc column refer to group labels: ^1^ = Control, ^2^ = colitis, ^3^ = Dexamethasone, ^4^ = OT 0.5 mg/kg, ^5^ = OT 1 mg/kg Symbols ‘<’ and ‘>’ indicate statistically significant differences between groups based on Dunn’s post hoc test.

**Table 6:** Results of serum biochemical parameter analysis.

Parameter	^1^ Control n = 8	^2^ Colitis n = 8	^3^ Dexamethasone n = 8	^4^ OT 0.5 mg/kg n = 8	^5^ OT 1.0 mg/kg n = 8	*p*	Post Hoc (Dunn)
*Values presented as Mean ± SD (upper line) and Median (IQR) (lower line, italics)*							
**TNF-α** (ng/mL)	**178 ± 16** *(164–186)*	**168 ± 25** *(150–181)*	**156 ± 19** *(142–172)*	**174 ± 36** *(147–198)*	**183 ± 34** *(164–216)*	0.288	—
**IL-1β** (pg/mL)	**2146 ± 528** *(1696–2572)*	**2189 ± 408** *(2016–2525)*	**2104 ± 558** *(1589–2541)*	**1856 ± 289** *(1613–2063)*	**2135 ± 190** *(2025–2282)*	0.469	—
**IL-6** (ng/mL)	**7 ± 1** *(6–7)*	**5 ± 1** *(5–6)*	**6 ± 1** *(6–7)*	**6 ± 1** *(5–7)*	**7 ± 1** *(6–7)*	0.045 **	**2 < 1, 5**
**MPO** (ng/mL)	**19 ± 3** *(15–21)*	**3 ± 1** *(3–4)*	**28 ± 10** *(22–30)*	**28 ± 11** *(19–34)*	**36 ± 11** *(29–40)*	0.001 ***	**2 < 3, 4, 5; 5 > 1**
**MDA** (nmol/mL)	**4 ± 1** *(4–5)*	**5 ± 1** *(5–6)*	**5 ± 1** *(5–6)*	**2 ± 1** *(1–3)*	**2 ± 1** *(1–2)*	0.001 ***	**1, 2, 3 > 4, 5**
**GSH** (mg/L)	**712 ± 42** *(680–740)*	**744 ± 123** *(652–789)*	**752 ± 160** *(701–863)*	**821 ± 146** *(776–916)*	**898 ± 213** *(716–1048)*	0.113	—
**GPx** (U/mL)	**121 ± 18** *(103–137)*	**44 ± 20** *(23–60)*	**55 ± 35** *(24–89)*	**199 ± 84** *(146–233)*	**155 ± 23** *(140–170)*	0.001 ***	**1, 2 < 4, 5; 3 < 4, 5**
**SOD** (ng/mL)	**2 ± 0** *(2–2)*	**1 ± 1** *(0–2)*	**1 ± 1** *(0–3)*	**3 ± 2** *(2–4)*	**3 ± 0** *(3–3)*	0.003 ***	**2 < 4, 5; 3 < 4, 5**
**CAT** (ng/mL)	**82 ± 5** *(79–85)*	**75 ± 6** *(70–79)*	**79 ± 18** *(76–91)*	**69 ± 25** *(69–82)*	**98 ± 6** *(92–101)*	0.001 ***	**5 > 1, 2, 3, 4**

**OT:** Oxytocin; **TNF-α:** Tumor Necrosis Factor-alpha; **IL**: Interleukin; **MPO:** Myeloperoxidase; **MDA:** Malondialdehyde; **GSH:** Glutathione; **GPx:** Glutathione Peroxidase; **SOD:** Superoxide Dismutase; **CAT:** Catalase. Kruskal–Wallis test with post hoc Dunn test. ** *p* < 0.05; *** *p* < 0.01. Significant *p* values and post hoc comparisons are highlighted in bold. Numbers in the Post Hoc column refer to group labels: ^1^ = Control, ^2^ = colitis, ^3^ = Dexamethasone, ^4^ = OT 0.5 mg/kg, ^5^ = OT 1 mg/kg Symbols ‘<’ and ‘>’ indicate statistically significant differences between groups based on Dunn’s post hoc test.

**Table 7:** Evaluations of tissue-based measurements according to groups.

Parameter	^1^ Control n = 8	^2^ Colitis n = 8	^3^ Dexamethasone n = 8	^4^ OT 0.5 mg/kg n = 8	^5^ OT 1.0 mg/kg n = 8	*p*	Post Hoc (Dunn)
*Values presented as Mean ± SD (upper line) and Median (IQR) (lower line, italics)*							
**TNF-α** (ng/wet tissue)	**186 ± 34** *(168–205)*	**177 ± 47** *(146–222)*	**195 ± 49** *(159–218)*	**162 ± 52** *(129–204)*	**127 ± 45** *(100–143)*	0.043 **	**5 < 1, 2, 3**
**IL-1β** (pg/wet tissue)	**2007 ± 630** *(1676–2362)*	**1662 ± 735** *(925–2192)*	**2165 ± 758** *(1649–2456)*	**1939 ± 750** *(1204–2632)*	**1970 ± 599** *(1394–2378)*	0.737	—
**IL-6** (ng/wet tissue)	**8 ± 2** *(6–10)*	**7 ± 4** *(4–8)*	**13 ± 5** *(10–18)*	**6 ± 4** *(3–9)*	**6 ± 4** *(3–10)*	0.020 **	**3 > 4, 5**
**MPO** (ng/wet tissue)	**29 ± 6** *(23–35)*	**7 ± 4** *(4–9)*	**18 ± 24** *(4–25)*	**23 ± 15** *(10–35)*	**28 ± 9** *(22–33)*	0.009 ***	**2 < 1, 4, 5**
**MDA** (nmol/wet tissue)	**4 ± 1** *(3–4)*	**6 ± 2** *(5–7)*	**5 ± 1** *(4–6)*	**2 ± 1** *(1–2)*	**1 ± 1** *(1–2)*	0.001 ***	**1, 2, 3 > 4, 5**
**GSH** (mg/L/wet tissue)	**646 ± 68** *(595–674)*	**610 ± 136** *(513–727)*	**754 ± 219** *(592–925)*	**751 ± 146** *(620–849)*	**810 ± 131** *(732–886)*	0.045 **	**2 < 5**
**GPx** (U/wet tissue)	**53 ± 24** *(32–78)*	**37 ± 18** *(19–49)*	**114 ± 103** *(34–227)*	**137 ± 153** *(35–199)*	**178 ± 111** *(88–271)*	0.042 **	**2 < 4, 5**
**SOD** (ng/wet tissue)	**2 ± 1** *(1–3)*	**1 ± 2** *(0–1)*	**2 ± 2** *(1–4)*	**3 ± 3** *(1–4)*	**3 ± 2** *(1–5)*	0.490	—
**CAT** (ng/wet tissue)	**54 ± 21** *(46–65)*	**51 ± 15** *(43–62)*	**97 ± 21** *(86–103)*	**61 ± 34** *(29–82)*	**80 ± 34** *(56–106)*	0.004 ***	**3 > 1, 4; 2 < 3, 5**

**OT:** Oxytocin; **TNF-α:** Tumor Necrosis Factor-alpha; **IL:** Interleukin; **MPO:** Myeloperoxidase; **MDA:** Malondialdehyde; **GSH:** Glutathione; **GPx**: Glutathione Peroxidase; **SOD:** Superoxide Dismutase; **CAT:** Catalase. Kruskal–Wallis test with post hoc Dunn test. **p < 0.05; ***p < 0.01. Significant *p* values and post hoc comparisons are highlighted in bol. Numbers in the Post Hoc column refer to group labels: ^1^ = Control, ^2^ = colitis, ^3^ = Dexamethasone, ^4^ = OT 0.5 mg/kg, ^5^ = OT 1 mg/kg Symbols ‘<’ and ‘>’ indicate statistically significant differences between groups based on Dunn’s post hoc test.

## Data Availability

The data underlying this article are available in the article. If needed, please contact the corresponding author. The email address is aydinliburcu@gmail.com.
